# Percutaneous Endoscopic Lumbar Interbody Fusion: Technical Note and Preliminary Clinical Experience with 2-Year Follow-Up

**DOI:** 10.1155/2018/5806037

**Published:** 2018-11-19

**Authors:** Junlong Wu, Huan Liu, Shengxiang Ao, Wenjie Zheng, Changqing Li, Haiyin Li, Yong Pan, Chao Zhang, Yue Zhou

**Affiliations:** ^1^Department of Orthopaedics, The Second Affiliated Xinqiao Hospital of Army Medical University, Chongqing 400037, China; ^2^Department of Orthopaedics, Fourth Military of Chinese People Liberation Army, Xining, Qinghai 810007, China

## Abstract

**Objective:**

Endoscopic surgeries have been attempted in the field of lumbar decompression and fusion surgery in the past decade. Percutaneous endoscopic lumbar interbody fusion (PELIF) is a new-emerging technique taking advantages of an anatomical (Kambin's triangle) to achieve simultaneous decompression and fusion under endoscopic visualization. The purpose of this study is to evaluate the feasibility and safety of PELIF technique with general anesthesia and neuromonitoring.

**Methods:**

The authors present the details of PELIF technique with general anesthesia and neuromonitoring. The first 7 consecutive patients treated with minimum of 2 year's follow-up were included. Clinical outcomes were assessed by visual analog scale (VAS) for back and leg pain, Oswestry Disability Index (ODI) scores, and the Short Form-36 health survey questionnaire (SF-36) in the immediate preoperative period and during the follow-up period.

**Results:**

All patients underwent single-level PELIF surgery successfully and without conversion to open surgery. The average age was 56.0±13.0 years. All patients had Grade I degenerative/isthmic spondylolisthesis and 4 patients coexisted with disc herniation. The mean operative time was 167.5±30.9 minutes, and intraoperative blood loss was 70.0±24.5 ml. Postoperative drainage volume was 24.5±18.3 ml. The differences in the VAS scores for low back pain and leg pain between preoperative and follow-up were significant (P<0.05). The SF-36 Physical Component Summary (PCS) improved from 38.83±4.17 to 55.67±2.58 (P<0.001). The SF-36 Mental Component Summary (MCS) improved from 43.83±3.13 to 57.50±5.36 (P=0.001). The ODI score improvement rate was 33.7±3.7 %. All cases demonstrated radiopaque graft in the intervertebral disc space consistent with solid arthrodesis.

**Conclusions:**

PELIF technique seems to be a promising surgical technique for selected appropriate patients, with the minimal invasive advantages in decreased blood, shortage of ambulation time, and hospital stay, compared with MIS-TLIF. Because of limited Kambin's triangle space and the exiting nerve root nearby, PELIF is still a challenging technique. Future advancement and development in instrument and cage design are vital for application and popularization of this technique. Prospective, randomized, controlled studies with large sample size on PELIF technique are still needed to prove its safety, efficacy, and minimal invasive advantages.

## 1. Introduction

Conventional open posterior fusion surgery of the lumbar spine, though addressing the pathology adequately, may—depending on significant surgical destruction of posterior muscular-ligamentous complex—lead to muscular atrophy, postoperative back pain, and functional disability [1–4]. Therefore, several factors which include, but are not limited to, the desire to minimize complications and hospitalization; the desire to facilitate an early return to productive hospitalization; the desire for elderly patients to return to active premorbid status; and the desire to decrease the cost of medical care have combined to facilitate the paradigm shift from open to minimally invasive spine surgery (MIS) [5, 6].

Currently, there are many types of MIS lumbar fusion surgery, including transforaminal lumbar interbody fusion (TLIF), anterior lumbar interbody fusion (ALIF), extreme lateral lumbar interbody fusion (XLIF), and posterior lumbar interbody fusion (PLIF) [2, 7]. All these procedures, though sharing the label of MIS, have different attributes in terms of distraction of the normal anatomic structures; accessibility to the different levels of the spine [8]. The search for newer surgical methods to achieve the goals of minimally invasive surgery is essential.

Recently, endoscopic surgeries have been attempted in the field of lumbar decompression and fusion surgery [8–13]. Some of these techniques [9–11] are evolved from typical MIS-TLIF technique using smaller tubular retractor through wilts plane and endoscopy-assistance. In this study, we will mainly focus on percutaneous endoscopic lumbar interbody fusion technique (PELIF) based on full-endoscopic technique through Kambin's triangle, with a similar surgical access and manipulation as percutaneous endoscopic discectomy (PELD). This technique takes advantages of an anatomical corridor that allows for both decompression of the traversing and exiting nerve roots and approach to the interbody space in order to achieve simultaneous decompression and fusion under full-endoscopic visualization [14]. Meanwhile, the minimal invasive nature of this procedure may even allow surgery be performed without general anesthesia which might be great benefit decreasing anesthetic risk for elder patients [10]. The purpose of this article was to demonstrate the surgical technique of PELIF and share preliminary clinical experience.

## 2. Methods

This study is a retrospective analysis of a consecutive case series involving patients treated with endoscopic single-level PELIF at a single institution. All the medical records were anonymous, and no patient information was extracted except for research intention. All patients had Grade I degenerative/isthmic spondylolisthesis and 4 patients coexisted with disc herniation. A total 7 patients underwent follow-up for more than 30 months. Demographic characteristics, diagnosis, operation time, blood loss, drainage volume, time to ambulation, postoperative hospitalization days, and perioperative complications were evaluated. Clinical outcomes such as visual analog scale (VAS), Oswestry Disability Index (ODI) score, and the Short Form-36 health survey questionnaire (SF-36) were assessed before and after therapy. Postoperative complications and symptom recurrence requiring reoperation were assessed through review of medical record documentation and/or telephone interviews with patients. Fusion was considered to have occurred if the trabecular bone had been bridged, as seen on a postoperative CT scan.

### 2.1. Surgical Management and Technique

The patient is placed in prone position and the C-arm should be placed on the contralateral side of PELIF access. The patient's position on the table was adjusted to facilitate the disk approach, especially at level L5-S1, by increasing forward hip flexion but avoiding a kyphotic correction of the lumbar lordosis. In this case series, the authors used a percutaneous endoscopic technique for interbody fusion combined with screw fixation with general anesthesia and neuromonitoring. Lower extremity somatosensory evoked potential, transcranial electrical stimulation motor evoked potential, and spontaneous electromyography (EMG) was used to monitor nerve root function. The PELIF® O-Cage (Joimax GmbH, Germany) used in this procedure consists of an MRI-compatible titanium alloy (Ti6Al4V ELI) with osteoconductive surface which forms a base for optimal cell growth. The diamond cell structure increases the cage surface area and leads to optimal bony ingrowth. It is necessary to mention that PELIF® O-Cage is not designed as a “stand-alone” implant. The fusion should always be accompanied by posterior fixation of percutaneous pedicle screws and/or transarticular screws.

**Figure 1:**
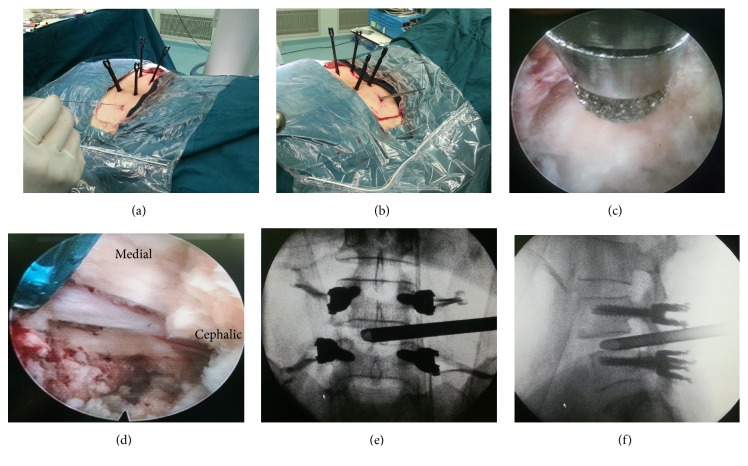
(a) Percutaneous transforaminal puncture into disk after percutaneous pedicle screw fixation. (b) Sequential dilation. (c) Optional foraminoplasty and expansion of the safety triangle by bone drill under endoscopic views. (d) Neurological decompression and initial endplate preparation in endoscopic view. (e) and (f) Working tube insertion in anteroposterior and lateral X-ray views.

Traditional transforaminal puncture of an 18G needle is carried out with the entry point between 8 and 14 cm (10-12 cm at L4/5) lateral to the spinous process at a 40° to 60° angle and as parallel to the intervertebral disc space as possible (Figure 1(a)), Axial MRI and CT images can be useful to design the needle trajectory and calculate the distance of the skin entry point away from the midline. The 18G needle is advanced into the intervertebral disc space; the style is removed; and a 0.8 mm guide wire is inserted through the cannula. Subsequent tissue dilation and bone resection by subsequent reamers is performed up to the diameter of the TESSYS® working tube as traditional PELD procedure (Figure 1(b)). Neurological decompression and optional foraminoplasty by bone drill/endoscopic burr can be performed if needed (Figure 1(c)). The annulus is opened and a primary disc removal and nerve root decompression is performed under endoscopic views (Figure 1(d)). Appropriate position of working tube insertion was confirmed with anteroposterior and lateral X-ray views (Figures 1(e) and 1(f)).

**Figure 2:**
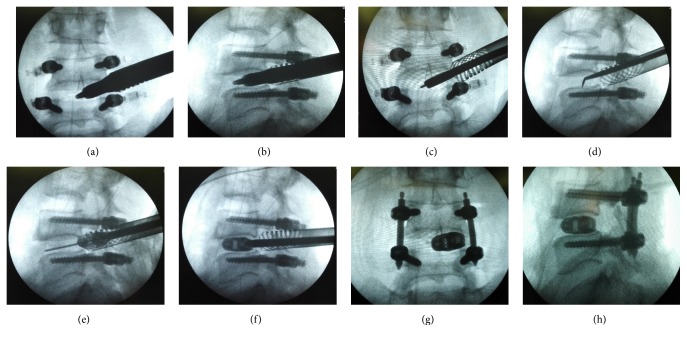
(a) and (b) Performing the dilation with the PELIF dilators until the desired diameter of the working tube. (c) and (d) Further removing intervertebral disc tissues and adequately endplate preparation. (e) and (f) Utilizing the guide wire to ease the cage placement under X-ray control. (g) and (h) Identification of the implant position by anteroposterior and lateral views.

The TESSYS® working tube is withdrawn, with a flexible 2.0 mm guide wire which is placed in the disc space instead. All instruments as well as the O-Cage itself can be perfectly positioned utilizing this guide wire. Perform the dilation with the PELIF® dilators until the desired diameter of the working tube (15 or 18 mm diameter) is achieved. The working tube is advanced over the dilators with a twisting motion counterclockwise until bone contact with the vertebral bodies. Subsequently, the working tube is anchored with a clockwise rotation onto the vertebrae and into the soft tissue (Figures 2(a) and 2(b)). The dilators are removed from the working tube. Placement of the endoscope adapter on the working tube in order to further remove intervertebral disc tissues under endoscopic view. If necessary, expanding the access using the bone drills (7.5 mm and 8.5 mm) to intervertebral disc space is extended to enable easier implantation of the cage. The raspatory is positioned between the end plates by using the 2.0 mm wire as a guide. The raspatories with different size are used sequentially for preparing the end plates by repeated rotation for at least 90°. The raspatories are also used for determining implant size under fluoroscopic control (Figures 2(c) and 2(d)). After fusion site preparation adequately, autogenous bone graft from superior articular process and commercial cancellous bone allograft was placed anteriorly and contralateral to the annulotomy within the interbody space through funnel-shaped bone graft device and the nerve root was again examined to ensure adequate decompression. Up to 35° degrees of cage angulation can be achieved by adjusted the distal knob of insertion instrument to ease the cage placement. The cage is then introduced into the intervertebral disc space trough the working tube by gently tapping on the back of the instrument handle under X-ray control, ideally with the 2.0 mm guide wire kept in place. Neurological feedback from neuromonitoring should be carefully watched during this section. Release the cage from connected instruments when it is in appropriate position (Figures 2(e) and 2(f)).

Check the implant position, the working tube is removed by turning it counterclockwise. (Figures 2(g) and 2(h)) Percutaneous pedicle screws are then finally compressed and locked. After all instruments were removed, a subfascial hemovac is inserted and direct closure of the skin was done. Postoperative management is similar with MIS-TLIF surgery, while earlier ambulation in the same day of surgery is encouraged and permitted with lumbar orthosis because of less bony removal and soft tissues injury [15, 16]. Drainage catheter is suggested in some studies to prevent postoperative hematoma because pressure of saline irrigation may lead the surgeon to overlook the potential epidural bleeding [12]. The patients are normally discharged 1 or 2 days after the surgery.

### 2.2. Statistical Analysis

The paired t test was performed for the preoperative and follow-up parameters (VAS, ODI, SF-PCS, and SF-MCS). The descriptive assessments and analytical statistics were performed depending on the group characteristics with SPSS (version 21.0, SPSS, Chicago, IL, USA). A positive significance was defined as probability of less than 0.05 for two sides.

## 3. Results

The demographic and baseline characteristics of the enrolled patients are shown in Table 1. The average age was 56.0±13.0 years (range 33-72 years). All patients had Grade I degenerative/isthmic spondylolisthesis and 4 patients coexisted with disc herniation. All patients underwent a single-level PELIF surgery successfully and without conversion to open surgery. Neurologic improvements were evident after surgery and persisted during the follow-up period. The mean operative time was 167.5±30.9 minutes (range 135-220 minutes), and intraoperative blood loss was 70.0±24.5 ml (rang 50-100 ml). Postoperative drainage volume was 24.5±18.3 ml (range 5-50 ml). The mean length of time to ambulation was 1.2±0.6 nights.

The preoperative clinical outcome assessments were respectively compared with postoperative 1 year and 2-year follow-up. All patients were tracked with 35.1±3.0 months mean follow-up (range 31.5-38.1 months). The differences in the VAS scores for low back pain and leg pain between preoperative and 1/2-year follow-up were significant (P<0.05). The SF-36 Physical Component Summary (PCS) improved from 38.83±4.17 to 55.67±2.58 (P<0.001). The SF-36 Mental Component Summary (MCS) improved from 43.83±3.13 to 57.50±5.36 (P=0.001). The ODI score improvement rate was 33.7±3.7 %. (Table 2)

**Figure 3:**
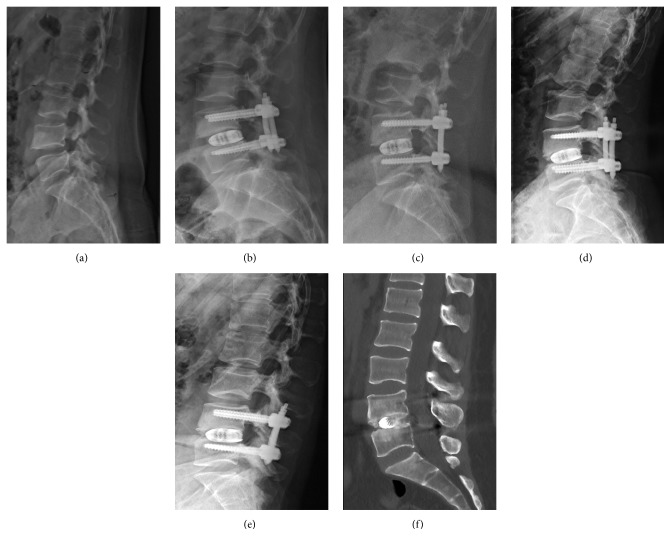
(a) Preoperative lateral radiograph showing isthmic spondylolisthesis. (b) and (c) Lateral radiograph at 1 and 12 months postoperative. (d) and (e) Extension and flexion lateral X-rays at two-years follow-up. (f) Sagittal CT image obtained 2 years postoperatively showing interbody fusion.

Radiographic imaging included flexion-extension radiographs and CT images were taken at 1, 12, and 24 months after surgery (Figure 3). All cases demonstrated radiopaque graft in the intervertebral disc space consistent with solid arthrodesis. There were no clinical or radiographic signs of nonunion. And there were no cases with perioperative and postoperative complication, such as dural tears, infection, or implant loosening. Revision surgery was not required in any patient.

## 4. Discussion

PELIF technique is a new-emerging technique evolved from PELD surgery in the recent decade; PELIF conducts lumbar interbody fusion through percutaneous transforaminal endoscopic access in Kambin's triangle like traditional PELD techniques [17]. PELIF were performed through sequential dilatation in soft tissues and very few bone removals compared with MIS-TLIF and theoretically offer advantages of less invasive, decreased blood loss, shorter patient recovery time, and the possibility of performing the surgery without anesthesia [8, 10, 16]. In this study, we demonstrated the feasibility and safety of PELIF technique with general anesthesia and shared clinical experiences with 2-year follow-up. Under general anesthesia, we found very little nerve distraction according to the method of progressive dilatation. From the anatomical perspective, the exiting root forms the hypotenuse of the working zone. The mean shortest distance between the root and facet surface was reported less than 2 mm at the upper disc margin level and less than 7 mm at the lower disc margin level [18]. Therefore, partial facetectomy of superior articular process is an essential step to provide us the sufficient space for PELIF procedures and eliminate exiting root injury [14]. So local anesthesia with/without sedation, low-dose epidural anesthesia, would be better choice for standard PELIF technique. Possibility of local anesthesia offers additional benefit for elder patients especially with systemic diseases.

### 4.1. Indications of PELIF Include the Following

Single-level fusion surgery from L3–4 to L5–S1 is initially recommended. Indications of PELIF were usually advised for degenerative disc disease, degenerative/isthmic spondylolisthesis, and spinal stenosis with instability. Postoperative instability or fail back syndrome (FBSS) to the lumbar spine is also an indication.

### 4.2. Contraindications Include, but Are Not Limited to the Following

Any condition which eliminates the potential profile of a spinal implant is relative contraindications, such as congenital abnormalities, bone resorption, osteopenia, poor bone quality and osteoporosis, infection, spondylodiscitis or signs of local inflammation, vertebral fractures, extremely narrow Kambin's triangle due to collapsed foramen/intervertebral disc height, or neurological abnormity; severe central stenosis could not be satisfactorily decompressed under PELD, high-grade spondylolisthesis.

Although only a few studies with small sample size have reported surgical technique and clinical results of PELIF, nearly all of the existent clinical studies [8, 10–13, 15, 16, 19] reported significant minimal invasive advantages superior to MIS-TLIF (e.g., smaller incision from 7-15mm, very early standing and ambulation at the same day of surgery with no additional care, and a significant reduced hospital stay). In contrast, posterior MIS-TLIF was reported to need an incision about 30 mm and splitting of paravertebral muscles; also the time after surgery until ambulation and hospital discharge may be up to 3.2 days and 9.3 days on average, respectively [20]. In the present study, the mean operative time was 167.5 minutes, and intraoperative blood loss was 70.0 ml. Postoperative drainage volume was just 24.5±18.3 ml. The mean length of time to ambulation was 1.2±0.6 nights. Through the expanded safety triangle zone approach, we can expose only the exiting nerve root to perform interbody fusion without intra-abdominal dissection or exposing central dura and traversing nerve root. No general complications include DVT and pulmonary embolism was reported. Other complications such as CSF leak and postoperative hematoma were seldom observed [8, 9, 21]. In our clinical practice, perioperative complication was also not observed. And the anesthesiologic risk may be eliminated; even local anesthesia is optional [19].

In preliminary practice of PELIF, stand-alone B-Twin expandable spacer is a common option of disc spacers [19, 20]. The small size of B-Twin expandable spacer facilitated its placement in a very small incision and working tube with minimal risk of neurological impairment. Disc height restore was satisfactory from preoperative 8.3±1.6 mm (range, 5.2–11.5) improved to 11.4±1.8mm (range, 8.8–14.7) in early postoperative period. However, excellent or good results were only obtained in only 72.2% of the patients which the author personally contributes it may because of a small sample size. Other literatures of percutaneous LIF studies using the B-Twin expandable spacer reported satisfactory results, but radiological results including disc space subsidence in all and breakage of implant limbs in some patients make the stand-alone application of the expandable spacer (without any posterior fixation) debatable [22]. In our study, the unexpandable O-Cage (Joimax GmbH, Germany) which consists of an MRI-compatible titanium alloy (Ti6Al4V ELI) with osteoconductive surface forms a base for optimal cell growth was used in the PELIF surgery. O-Cage is not designed as a “stand-alone” implant, so fusion should always be accompanied by posterior fixation of percutaneous pedicle screws or transarticular screws. As O-Cage is not an expandable cage, we just cautiously selected the appropriate patients except for extremely small Kambin's triangle area due to collapsed foramen/intervertebral disc height, severe central stenosis which could not be satisfactorily decompressed under PELD.

In 2013, Frederic Jacquot reported [23] the largest case series of PELIF with 57 patients and gave negative opinion for this technique. The author utilized rigid cage placement with stand-alone cages in 46 cases and contemporary posterior plate fixation in 11 patients. While extremely high cage migration and reoperation rate was reported in this trial, with 2 asymptomatic migration of the cages occurred required no further operation, 13 symptomatic migration (22.8 %), requiring a conventional secondary reoperation, after a mean delay of eight months (range three to 36 months) with no neurological deficit. Meanwhile, eight additional patients (14 %) suffered from postoperative paresis and painful syndromes. The author also mentioned that rest patient without above complications had excellent results following a very fast recovery and a very short hospital stay. The author concluded that PELIF technique is not recommended in its current state because of extremely high complication rate except technical improvements despite a prominent fast recovery. We suspected that an extremely high complication rate of cage migration and postoperative paresis compared with other PELIF reports may be related to the following intraoperative factors although detailed surgical procedures were not given: inadequate disc preparation due to very fast surgery and calcium phosphate substitute filled in cages with no autograft or other alternatives prefilled in disc space before cage insertion mentioned, nonexpandable stand-alone cages were used and no foraminoplasty was reported to employ in this clinical trial, in addition, a considerable lager number of patients were operated in upper lumbar segment with anatomical narrow Kambin's triangle. In this study, all patients underwent a single-level PELIF surgery successfully and without conversion to open surgery. Neurologic improvements were evident after surgery and persisted during the follow-up period. Two-year follow-up showed significant improvement in VAS, ODI score, SF-36 PCS, and MCS, which were consistent with the previous studies [10]. Fusion was obtained in all cases with radiopaque graft in disc space consistent with solid arthrodesis and no clinical or radiographic signs of nonunion.

A thorough understanding of foraminal anatomy is fundamental for considering how to safely access the disc space and what shapes and sizes of interbody implants are feasible for use in the foramen [14]. Considering stand-alone cages may increase the risk of migration and/or subsidence, when compared to cage fusion with additional pedicle screw fixation, some of the recent studies trended to applied additional percutaneous pedicle screw and/or transarticular screw [16]. Self-expandable cage design seems to be better option for PELIF technique as related literature described. Firstly, self-expandable cage which has smaller initial size facilitates cage insertion and reduces possible neurological invasion [19, 24]. Study of Rudolf Morgenstern indicated [16] improvement of leg pain was slightly higher in patients treated with the expandable cage than in patients treated with the PEEK cage. Other possible advantages were also mentioned as follows: expandable cages allow indirect neural decompression and additional foraminal expansion by restoring intervertebral height; immediate stability to the fixation construct was also enhanced. In cases of spondylolisthesis, percutaneous expanded interbody implants may offer convenient distraction and reduction.

Exiting root injury presented as postoperative paresis and radical pain is specific and common complication for pTLIF technique similar but more common than PELD because more occupation of transforaminal space due to cage insertion. Rudolf Morgenstern [16] suggested neuromonitoring to be routinely performed in general anesthesia with somatosensory evoked potentials (SEP) and motor evoked potentials (MEP) be employed during the whole surgical procedure to monitor all involved peripheral nerves. Additional nerve stimulation was also performed to ensure that nerve roots were not compromised at special conditions such as cage insertion. A bevel-end working tube should be use and careful rotation of the bevel may be helpful for protection of the exiting root during procedure. Foraminoplasty is always necessary especially at the level of L5-S1 or any situation needed [19, 21]. In addition, more reliability and efficiency endoscopic approaches which access the inferior disc space-superior endplate junction at the medial wall of the pedicle can achieves exponential (*π*r2) increases in disc space dilation for interbody implant placement and decrease nerve root distraction [14].

Despite all the benefit above mentioned, PELIF seems to be an immature and high-demanding and controversial procedure with limited indication and possible specific complications. Very narrow space of Kambin's triangle cause technique difficulties for thorough disc preparation and safe cage insertion, leading to complications like exiting nerve root injury, nonunion, or cage migration. Other obstacles included steep learning curve, need for rich full-endoscopic experience, lack of autograft due to few bone removal, excessive radiation exposure increases fear of for the patient, and the surgical team. Finally, it is essential to point out that all of the related several studies on PELIF technique were preliminary retrospective, uncontrolled trails with relatively small sample size, which make us incapable to give a comprehensive and definitive assess on it at present.

## 5. Conclusions

Present PELIF technique with the titanium alloy spacer seems to be a promising surgical technique for selected appropriate patients. The clinical results of attempt in PELIF technique support the minimal invasive advantages in decreased blood, shortage of ambulation time, and hospital stay, compared with MIS-TLIF. Steep learning curve with rich previous PELD experience needed. Because of limited Kambin's triangle space, PELIF technique is still a challenging procedure. Future advancement and development in instrument and cage design are vital for application and popularization of this technique. Prospective, randomized, controlled studies with large sample size on PELIF technique are still needed to prove its safety, efficacy, and minimal invasive advantages.

## Figures and Tables

**Table 1:** Clinical summary of enrolled patients.

Case No.	Sex/ Age (y)	Duration of Disease (months)	Operation Time (min)	Blood Loss (ml)	Drainage Volume (ml)	Follow-up Time (months)	Operative Level
1	M/57	6	220	100	40	38.1	L4/5
2	F/59	36	165	100	50	37.9	L4/5
3	F/33	24	145	50	12	37.5	L4/5
4	F/53	120	185	50	5	33.0	L4/5
5	M/62	84	135	50	10	32.7	L4/5
6	M/72	36	155	70	30	31.5	L4/5

**Table 2:** Preoperative, follow-up VAS, ODI, and SF-36 scores.

Characteristics	Value	P value
Lower back pain VAS, mean ± SD		
Preoperative	6.17±0.75	-
Postoperative 1 year	0.83±0.75	<0.001_ _^*∗*^
Postoperative 2 years	0.67±0.52	<0.001_ _^#^
Lower extremity pain VAS, mean ±SD		
Preoperative	5.33±1.97	-
Postoperative 1 year	0.33±0.52	0.004_ _^*∗*^
Postoperative 2 years	0.17±0.41	0.002_ _^#^
SF-36 PCS, mean ± SD		
Preoperative	38.83±4.17	-
Postoperative 1 year	51.33±3.20	<0.001_ _^*∗*^
Postoperative 2 years	55.67±2.58	<0.001_ _^#^
SF-36 MCS, mean ± SD		
Preoperative	43.83±3.13	-
Postoperative 1 year	56.33±6.83	0.009_ _^*∗*^
Postoperative 2 years	57.50±5.36	0.001_ _^#^
ODI score, mean ± SD		
Preoperative	44.83±4.75	-
Postoperative 1 year	14.50±8.09	<0.001_ _^*∗*^
Postoperative 2 years	11.17±4.31	<0.001_ _^#^

*∗* p<0.05, postoperative 1 year compared with preoperative.

# p<0.05, postoperative 2 years compared with preoperative.

VAS, visual analog scale; MCS, Mental Component Score; PCS, Physical Component Score; SF-36, Short Form-36 Health Surgery Questionnaire.

## Data Availability

The data used to support the findings of this study are available from the corresponding author upon request.
